# Identification of miRNA-mRNA regulatory modules by exploring collective group relationships

**DOI:** 10.1186/s12864-015-2300-z

**Published:** 2016-01-11

**Authors:** S. M. Masud Karim, Lin Liu, Thuc Duy Le, Jiuyong Li

**Affiliations:** School of Information Technology and Mathematical Sciences, University of South Australia, Mawson Lakes, Adelaide, 5095 SA Australia

**Keywords:** miRNA-mRNA regulatory modules, Collective group relationships, Group pair, Canonical correlations

## Abstract

**Background:**

microRNAs (miRNAs) play an essential role in the post-transcriptional gene regulation in plants and animals. They regulate a wide range of biological processes by targeting messenger RNAs (mRNAs). Evidence suggests that miRNAs and mRNAs interact collectively in gene regulatory networks. The collective relationships between groups of miRNAs and groups of mRNAs may be more readily interpreted than those between individual miRNAs and mRNAs, and thus are useful for gaining insight into gene regulation and cell functions. Several computational approaches have been developed to discover miRNA-mRNA regulatory modules (MMRMs) with a common aim to elucidate miRNA-mRNA regulatory relationships. However, most existing methods do not consider the collective relationships between a group of miRNAs and the group of targeted mRNAs in the process of discovering MMRMs. Our aim is to develop a framework to discover MMRMs and reveal miRNA-mRNA regulatory relationships from the heterogeneous expression data based on the collective relationships.

**Results:**

We propose *DIscovering COllective group RElationships* (*DICORE*), an effective computational framework for revealing miRNA-mRNA regulatory relationships. We utilize the notation of collective group relationships to build the computational framework. The method computes the collaboration scores of the miRNAs and mRNAs on the basis of their interactions with mRNAs and miRNAs, respectively. Then it determines the groups of miRNAs and groups of mRNAs separately based on their respective collaboration scores. Next, it calculates the strength of the collective relationship between each pair of miRNA group and mRNA group using canonical correlation analysis, and the group pairs with significant canonical correlations are considered as the MMRMs. We applied this method to three gene expression datasets, and validated the computational discoveries.

**Conclusions:**

Analysis of the results demonstrates that a large portion of the regulatory relationships discovered by *DICORE* is consistent with the experimentally confirmed databases. Furthermore, it is observed that the top mRNAs that are regulated by the miRNAs in the identified MMRMs are highly relevant to the biological conditions of the given datasets. It is also shown that the MMRMs identified by *DICORE* are more biologically significant and functionally enriched.

**Electronic supplementary material:**

The online version of this article (doi:10.1186/s12864-015-2300-z) contains supplementary material, which is available to authorized users.

## Background

microRNAs (miRNAs) are a family of small (i.e. with typical length of 19–25 nucleotides) non-protein-coding RNA molecules that can play important regulatory roles in animals and plants [1, 2]. They base-pair with messenger RNAs (mRNAs) of protein-coding genes to induce mRNA degradation or translational repression [3]. The mature human miRNAs potentially target majority of the human mRNAs [4]. It has been demonstrated that miRNAs regulate a wide range of biological or cellular processes such as proliferation [5, 6], metabolism [7], differentiation [8], development [9], apoptosis [10], cellular signaling [11], and cancer development and progression [12–15].

There is a growing body of literature showing that multiple miRNAs are coordinated by forming cohesive groups to collectively regulate one or more pathways [16, 17]. The collective relationships yielded between a group of miRNAs and a group of mRNAs due to the tendency of the group formation act as a vital force in catering similar functioning miRNAs and mRNAs together. Therefore, the collective relationships between cohesive groups of miRNAs and their targeted mRNAs may provide better understandings on robust and potent regulatory relationships of miRNA-mRNA regulatory modules (MMRMs).

Several algorithms have been proposed to identify MMRMs from expression data using different approaches including Bayesian network learning [18], rule induction [19], association rule mining [20], population-based probabilistic learning [21], probabilistic graphical model [22–24], matrix factorization [25], and graph mining [17, 26]. Most of these existing methods do not consider the collective relationships between a group of miRNAs and the group of targeted mRNAs in the process of identifying MMRMs. In addition, many of them are either stochastic, or require prior knowledge such as number of modules to be identified, confirmed interactions, target site information [27].

Adapting a greedy overlapping neighborhood expansion clustering method, *ClusterONE*, which was developed to discover protein complexes from protein-protein interactions networks, Li et al. [27] proposed a clustering algorithm, *Mirsynergy* to detect synergistic miRNA regulatory modules. However, it requires and depends on the prior knowledge of confirmed gene-gene interactions. Recently Karim et al. [28] coined the notion of *collective group relationships*, and developed a method by integrating unweighted graphing mining concept and canonical correlation analysis to explore miRNA-mRNA regulatory relationships. However, it is noted that unweighted graph mining techniques are associated with limitation in representing the true interactions, and sometimes fail to capture correct regulatory relationships. Whereas weighted graph mining approaches can greatly improve the detection of the module structures [29], and hence regulatory relationships.

In this paper, we propose an effective computational framework, *DIscovering COllective group RElationships* (*DICORE*) to identify MMRMs and hence reveal miRNA-mRNA regulatory relationships from heterogeneous data. In order to extract MMRMs from the given gene expression datasets, we utilize the notion of collective group relationships, which provide MMRMs with additional quantitative strength information. The method finds a deterministic solution to the problem of discovering MMRMs from weighted bipartite graph representation of the given datasets, and rank the collective group relationships based on their strength of collective relationships. We apply *DICORE* to a dataset for Epithelial to Mesenchymal Transition, a breast cancer dataset, and a multi-class cancer dataset. Based on the knowledge from the literature, it is observed that the identified MMRMs exhibit enriched functionality with biological significance.

## Methods

### Problem statement

Consider two sets of variables **X**={*X*_1_,…,*X*_*p*_} and **Y**={*Y*_1_,…,*Y*_*q*_} such that $\mathbf {X} \cap \mathbf {Y} = \varnothing $, representing the attributes of two different types of objects. In this paper, **X** and **Y** refer to the expression levels of a set of miRNAs and a set of mRNAs, respectively. With their given datasets, *D*_*X*_ and *D*_*Y*_, having *n* matching miRNA and mRNA expression samples, our goal is to identify any *C*_*x*_⊂**X** and *C*_*y*_⊂**Y**, such that *C*_*x*_ and *C*_*y*_ are related, as a result of miRNAs in *C*_*x*_ collaboratively interacting with mRNAs in *C*_*y*_ and vice versa. We call (*C*_*x*_,*C*_*y*_) a *group pair*, and the relationship between *C*_*x*_ and *C*_*y*_ a *COllective group RElationship* (in short, *CORE*). The COREs are characterized by both group pairs and the collective relationships among the two cohesive groups in group pairs. Then the group pair (*C*_*x*_,*C*_*y*_) is an MMRM if the strength of the CORE between *C*_*x*_ and *C*_*y*_ is significant.

In order to discover COREs, and thus to identify MMRMs, we develop a two stages method, *DIscovering CORE* (*DICORE*). Two measures, collaboration score and canonical correlations, are employed in the two stages respectively. In the following, we firstly overview the workflow of *DICORE*, and then present the details of *DICORE*, including the definition of the collaboration score and the calculation of canonical correlations.

### Overview of *DICORE*

**Fig. 1:**
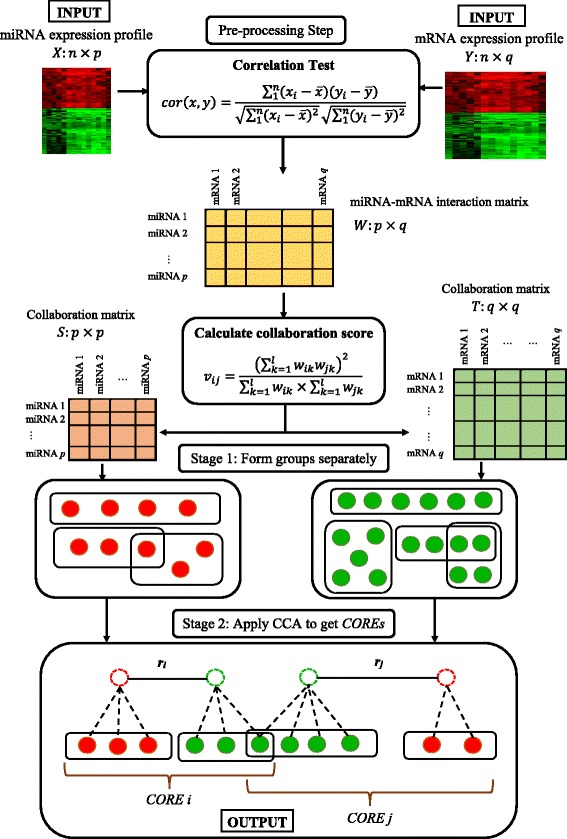
*DICORE* workflow. Given the inputs of miRNA and mRNA expression profiles, we first derive an expression-based interaction weights matrix *W* using correlation test. We then compute two collaboration score matrices *S* and *T* from *W* for miRNAs and mRNAs based on their functional interaction similarities with common mRNAs (or miRNAs), respectively. Using these collaboration scores as input, we separately generate groups of miRNAs and groups of mRNAs at Stage 1 by an overlapping neighborhood expansion clustering algorithm, in which miRNAs or mRNAs are greedily added to (removed from) each cluster of miRNAs or mRNAs, respectively that maximize cohesiveness score of the cluster. Next in Stage 2, we apply canonical correlation analysis on the groups of miRNAs and groups of mRNAs to obtain significant collective group relationships, which are eventually the MMRMs with strength scores

Figure 1 shows the workflow of *DICORE*. The overall workflow comprises a data pre-processing step and two main stages: (1) forming separate miRNA and mRNA groups and (2) searching for COREs.

In the data pre-processing step, *DICORE* first creates a weighted bipartite graph representation of the relationships among the individual variables of the given miRNA and mRNA expression profiles. Taking the variables as the vertices of a weighted bipartite graph *G*, a weighted edge is introduced between a miRNA variable and a mRNA variable to represent their interaction. Referring to Fig. 1, given *p* miRNAs and *q* mRNAs, let *W* denote the (*p*×*q*) miRNA-mRNA interaction weights matrix, where *w*_*ij*_ is the interaction weight for miRNA *i* targeting mRNA *j*. To compute miRNA-mRNA interaction weights, we calculate the *Pearson correlation coefficient* (*PCC*) [25] between each pair of miRNA and mRNA using the R built-in function, *cor*. The obtained PCCs are within the range of [−1,1], and the signed correlation coefficients provide two types of valuable information: the absolute values implying the strength of the miRNA-mRNA interactions (the higher the values, the stronger the interactions), and the signs indicating the directions of the associations. However, as the aim of the paper is to identify MMRMs (and thus to uncover miRNA-mRNA regulatory relationships), the collaboration score (explained in the next section) defined for discovering the modules considers the sum of the miRNA-mRNA correlations. In order to cater for both up and down miRNA regulations when calculating the total strength of the interactions, we use absolute values of the PCCs in the interaction weights matrix *W*, otherwise the signed PCCs or interaction weights will cancel out in Eq. (1).

Due to the higher possibility of dense interactions in the expression profile datasets, complete weighted graph mining may not be able to distinguish correct group structure. Accordingly we used a cutoff threshold *η* to trade off between the two extreme approaches namely complete unweighted graph mining and complete weighted graph mining.

At stage 1, we separately identify groups of miRNAs and groups of mRNAs. Referring to Fig. 1, based on the interaction weights matrix *W*, we firstly calculate the collaboration score between each pair of miRNAs and create the miRNA-miRNA collaboration matrix, *S*. The collaboration score between a pair of miRNAs reflects their similarity or collaboration in regulating target mRNAs (more details of collaboration scores are given in the next section). In a similar way, we compute the collaboration score between each pair of mRNAs (which implies their similarity in being regulated by miRNAs) and create the mRNA-mRNA collaboration matrix, *T*.

The identification of groups of miRNAs (or groups of mRNAs) is formulated as an overlapping clustering problem. Only the miRNAs (or mRNAs) that have strong collaboration between them are put in the same group, i.e. we use their collaboration scores as the similarity measure for the clustering. The clustering process is then aimed at maximizing the overall similarity of the miRNAs (or mRNAs) within the same group. We define such overall similarity within a group as the cohesiveness of a group (details of the definition is provided in the next section). The underlying clustering algorithm adapts from *ClusterONE*, which was originally developed for protein protein interaction networks [29]. Adopting the idea from [25], we discard groups with fewer than 5 mRNAs (i.e. minimum size threshold for mRNAs, *θ*_*g*_=5), as they usually do not provide relevant information. Similarly, we are not interested to consider groups having more than 500 mRNAs. Additionally, in order to avoid ‘star-shaped’ basic network structure, we choose 3 as minimum size threshold for miRNAs, *θ*_*m*_.

At stage 2, we use canonical correlation analysis to compute the strength of the collective relationships between groups of miRNAs and groups of mRNAs in terms of *canonical correlations*, and obtain COREs, which is eventually equivalent to MMRMs with additional quantitative information. We considered only the top COREs identified (i.e. the COREs with the higher canonical correlations), having minimum canonical correlation of *ρ*=0.50.

### Details of *DICORE*

In the following, we introduce the details of the collaboration score and how CCA is used to measure the strength of the collective group relationships.

The *collaboration score* expresses the degree of collaboration between two miRNAs (or between two mRNAs) considering their common interactions with mRNAs (or miRNAs). Given miRNA *i*, miRNA *j* (≠*i*) and the interaction weights matrix *W*, the collaboration score of the two miRNAs is calculated as follows:

(1) $$  v_{ij} = \frac{\left(\sum\limits_{k=1}^{l} w_{ik}w_{jk}\right)^{2}}{\sum\limits_{k=1}^{l} w_{ik} \times \sum\limits_{k=1}^{l} w_{jk}},  $$

where *l* is the number of other possible components that both miRNA *i* and miRNA *j* interact with, in this case mRNAs, so *l*=*q*. Let *S* refer to the miRNA-miRNA collaboration matrix of size *p*×*p*, where *s*_*ij*_=*v*_*ij*_.

Similarly, we compute the mRNA-mRNA collaboration score between mRNA *i* and mRNA *j* (≠*i*) by applying Eq. (1) on the transpose of the interaction weights matrix *W*, where *l*=*p*, the number of miRNAs. Let *T* refer to the mRNA-mRNA collaboration matrix of size *q*×*q*, where *t*_*ij*_=*v*_*ij*_.

Notably, if *W* were a binary matrix, Eq. (1) became the ratio of number of target mRNAs shared between miRNA *i* and miRNA *j* over the numbers of target mRNAs possessed separately by miRNA *i* or miRNA *j* (or the ratio of number of common miRNAs regulate both mRNA *i* and mRNA *j* over the numbers of miRNAs individually regulate mRNA *i* or mRNA *j*). An miRNA (or an mRNA) *i* is then ranked by the total collaboration score as $\sum \limits _{k=1}^{p}s_{\textit {ik}} \left (\text {or} \sum \limits _{k=1}^{q}t_{ik }\right)$.

Using collaboration scores as the similarity measures of pairs of miRNAs or pairs of mRNAs, miRNAs and mRNAs are clustered separately into cohesive groups by using a greedy strategy that maximize the cohesiveness score of groups. Similar to the cohesiveness defined in [29], we define *cohesiveness* score, *cs*(*C*_*i*_) for any group *C*_*i*_ as follows:

(2) $$  cs(C_{i}) = \frac{w_{int}(C_{i})}{w_{int}(C_{i}) + w_{ext}(C_{i}) + \alpha * |C_{i}|}  $$

where *w*_*int*_(*C*_*i*_) denotes the sum of the collaboration scores of all the internal pairs of variables, i.e. each pair only contains variables within the group *C*_*i*_; *w*_*ext*_(*C*_*i*_) is the sum of the collaboration scores of all the external pairs, i.e. each pair contains one variable within the group *C*_*i*_ and one variable outside the group *C*_*i*_; and *α*∗|*C*_*i*_| is a penalty term asserting the existence of unidentified interactions in the dataset, practically assuming that every component in *C*_*i*_ has *α* additional interactions that are undetected due to the limitations in the experimental setting.

*DICORE* uses *canonical correlation analysis* (*CCA*) [30] to compute the strength of the collective relationships between a group of miRNAs and a group of mRNAs in terms of the group pair’s canonical correlations. CCA is commonly used for quantifying the linear association between two sets of variables. Consider $\mathcal {A} = \vec {a}'\vec {X},\,\mathcal {B} = \vec {b}'\vec {Y}$ be the corresponding linear combinations of sets of variables $\vec {X}$ and $\vec {Y}$ respectively, where $\vec {a}$ and $\vec {b}$ are coefficient vectors. Vectors $\vec {a}$ and $\vec {b}$ are chosen such that the correlation between $\mathcal {A}$ and $\mathcal {B}$, i.e.,

(3) $$  \mathbf{r} = Corr(\mathcal{A}, \mathcal{B}) = \frac{\vec{a}'\Sigma_{XY}\vec{b}}{\sqrt{\vec{a}'\Sigma_{XX}\vec{a}}\sqrt{\vec{b}'\Sigma_{YY}\vec{b}}}  $$

is maximized, where *Σ*_*XX*_, *Σ*_*YY*_ and *Σ*_*XY*_ are variance of $\vec {X}$, variance of $\vec {Y}$, and covariance between $\vec {X}$ and $\vec {Y}$, respectively. The correlation **r** between the pair of linear combinations in Eq. (3) is called *canonical correlation*. Specifically, canonical correlation between a group of miRNAs and a group of mRNAs is computed using the R function *CCA* from the package PMA.

The intuition behind applying CCA is twofold. Firstly CCA captures weight scores of all interactions between all miRNAs and mRNAs in both groups of a group pair, while computing the strength of the collective interactions of the group pair. As a consequence, CCA mitigates the loss of weight scores of interactions due to the application of cutoff threshold *η* earlier. Secondly, it also makes it possible for a group of miRNAs (or a group of mRNAs) to be included in more than one CORE i.e. one module, if the strength of collective interactions satisfies the specified threshold.

### Data collection

Three real-world gene expression datasets are used to validate *DICORE*: an NCI60 dataset for Epithelial to Mesenchymal Transition, a breast-cancer (BR) dataset, and a multi-class cancer (MCC) dataset. The pre-processed differentially expressed gene expression datasets were collected from [31].

Epithelial to Mesenchymal Transition (EMT) is a biological process that enables cells to acquire migratory mesenchymal characteristics by losing epithelial features. The EMTs are associated with embryonic development, wound healing, organ fibrosis, and in the initiation of metastasis for cancer progression. The NCI60 dataset includes 60 cancer cell lines from the National Cancer Institute (NCI). Cell lines categorized as epithelial (11 samples) and mesenchymal (36 samples) were used for this work. As a result of the differential gene expression analysis, 1154 mRNAs and 35 miRNAs were identified to be differentially expressed at significant level (adjusted *p*-value <0.05, adjusted by Benjamini-Hochberg (BH) method).

The BR dataset includes expression profiles of the 50 cell lines of breast cancer. The cell lines were categorized as luminal (27 cell lines) and basal (23 cell lines). In the dataset, 89 miRNAs (adjusted *p*-value <0.02) and 1500 mRNAs (adjusted *p*-value <0.0001) were identified to be differentially expressed.

The MCC dataset includes samples from multiple cancers namely bladder, breast, colon, kidney, lung, pancreas, prostate and uterus. Samples of the dataset classified as normal (21 samples) and tumor (67 samples) were used in this work. In total, 62 miRNAs and 1318 mRNAs were obtained to be differentially expressed at significant level (adjusted *p*-value <0.05).

The datasets are available in Additional file 1.

We used the expression data to calculate the miRNA-mRNA interaction weights matrix *W*. We obtained the interaction weights of *W* by computing the absolute values of the Pearson correlation coefficients between pairs of miRNA and mRNA.

In order to obtain the ‘ground-truth’ databases of experimentally confirmed miRNA-mRNA interactions, we combined the interactions from four popular interactions databases, namely DIANA-TarBase v7.0 [32], miRTarBase v4.5 [33], miRecords v2013 [34], and miRWalk v2.0 [35]. While miRWalk contains both predicted and experimentally validated miRNA-mRNA interactions, rest of the databases include high quality manually curated experimentally validated miRNA-mRNA interactions published in the literature. Recently published DIANA-TarBase v7.0 alone included more than half a million interactions utilizing cell types from 24 species. We also added a HITS-CLIP database [36], which lists the confirmed targets of two miRNAs, namely *miR-200a* and *miR-200b*. We extracted only the confirmed miRNA-mRNA interactions associated with the human miRNAs and mRNAs given in the input datasets, and removed the duplicate entries. Finally, we obtained ‘ground-truth’ databases of 2147, 5791, and 8733 unique miRNA-mRNA interactions for the 29 miRNAs in the NCI60 dataset (there are no confirmed interactions for the 6 miRNAs with the name prefix *hsa-miRPlus-*), 89 miRNAs in the BR dataset, and 62 miRNAs in the MCC dataset, respectively. Details of the ‘ground-truth’ databases are available in Additional file 2.

## Results and discussions

We ran the experiment for all values for the cutoff threshold *η* in the range from 0 to 1 with a step size of 0.05. We only reported the summary and top results for each dataset. In each summary table, *#C*, $\overline {mR}$, $\overline {miR}$, $\bar {r}$, and *t* denote the number of COREs identified, average number of mRNAs in COREs, average number of miRNAs in COREs, average strength of the COREs, and time taken for the execution in seconds, respectively. The group distributions and all COREs for all datasets are described in details on our website (visit [37]).

### The NCI60 Dataset

The results obtained from the NCI60 dataset are summarized in Table 1. It is clear from the summary that potentially interesting results are obtained for the *η* values ranging from 0.60 to 0.85. By lowering the values of *η*, more miRNAs and mRNAs were added to these groups. For more in-depth analysis, we look more closely at some of the particular results.

**Table 1:** Summary of results of *DICORE* on the NCI60 dataset

*η*	*#* *C*	$\overline {mR}$	$\overline {miR}$	$\bar {r}$	*t*
0.45	1	6.00	35.00	0.61	1142.88
0.50	4	8.50	32.00	0.64	635.31
0.55	3	11.67	27.00	0.69	246.17
0.60	8	57.00	19.00	0.80	80.89
0.65	6	83.67	10.50	0.79	86.95
0.70	4	107.50	6.25	0.81	24.30
0.75	4	44.00	5.00	0.87	4.78
0.80	2	22.50	5.00	0.91	2.44
0.85	1	12.00	5.00	0.95	0.90

We obtained the most informative result (in terms of the strength of COREs, and number of experimentally confirmed interactions covered) for *η*=0.60, with 8 COREs involving 1 miRNA groups and 8 mRNA groups. The only group of miRNAs ‘m1N60’ catered in total 19 miRNAs including the *miR-200* family. On the other hand, we got the top mRNAs group (group having highest cohesiveness) ‘g1N60’ having 348 mRNAs. It included *CDH1* (epithelial cadherin or in short E-cadherin, a classical member of the cadherin superfamily, which plays a vital role in EMT such that EMT is also characterized by repression of E-cadherin expression), *ZEB1* (E-cadherin transcriptional repressor, which is usually targeted by *miR-200* family), and *TWIST1* (one of the important EMT inducers).

Furthermore, another interesting result was obtained for *η*=0.65. We got 6 COREs from 2 groups of miRNAs and 3 groups of mRNAs. The top miRNAs group ‘m1N65’ catered 14 miRNAs and is a proper subset of ‘m1N60’. The second miRNAs group ‘m2N65’ included total 7 miRNAs including 3 miRNAs from the *miR-17* miRNA gene family, namely *miR-106b*, *miR-18a*, and *miR-18b*.

### The BR dataset

From the summary given in Table 2, it is seen that higher informative results were obtained for *η* values from 0.55 to 0.65 from the BR dataset. The most informative result was obtained for *η*=0.60. We got 33 COREs from 2 groups of miRNAs and 17 groups of mRNAs. The top group of miRNAs included *miR-221* and *miR-222*, both of them are known to play important regulation role in aggressive breast cancer [38].

**Table 2:** Summary of results of *DICORE* on the BR dataset

*η*	*#* *C*	$\overline {mR}$	$\overline {miR}$	$\bar {r}$	*t*
0.50	3	8.00	15.00	0.66	1938.13
0.55	44	23.57	6.18	0.63	2043.01
0.60	33	48.09	6.61	0.70	216.53
0.65	24	47.79	3.00	0.69	36.49

### The MCC dataset

Table 3 shows the results obtained by *DICORE* on the MCC dataset. The most informative result is obtained for *η*=0.45. It catered all members of both *let-7* and *miR-30* miRNA gene families into the top group of miRNAs along with some other similar functioning miRNAs.

**Table 3:** Summary of results of *DICORE* on the MCC dataset

*η*	*#* *C*	$\overline {mR}$	$\overline {miR}$	$\bar {r}$	*t*
0.40	7	15.71	35.00	0.58	354.30
0.45	10	84.70	20.40	0.58	540.36
0.50	5	30.40	29.00	0.72	12.40
0.55	1	55.00	25.00	0.80	4.18
0.60	1	19.00	9.00	0.82	1.45

**Table 4:** Confirmed interactions in COREs from the NCI60 for *η*=0.65

ID	Confirmed interactions
C1N65	**miR-141**: BICD2, CDH1, EHF, IRF6, KLF5,
	PARD6B, RAB32, RAB8B, RHOD, SLC20A1,
	TWIST1;
	**miR-148b**: BIK, DDR1, ELOVL5, ERMP1, FAM84B,
	KLF5, MAL2, MAP1B, QKI, RAB8B, ST14,
	TBC1D30, TRAF4;
	**miR-200a**: BICD2, CDH1, EHF, ELOVL5, GRHL2,
	ITGB4, MAP1B, MSN, PARD6B, RAB32, TWIST1;
	**miR-200b**: AP1S2, ARHGAP32, CDH1, CLDN4, DSP,
	ELOVL5, ENSA, EPCAM, ESRP2, KIAA1949, KLF5,
	MAL2, MAP1B, MAPK13, MSN, OSTM1, PARD6B,
	QKI, SACS, SLC20A1, TINAGL1, TTL, TWIST1;
	**miR-200c**: AP1S2, CDH1, ENSA, ICA1, MSN,
	OSTM1, PARD6B, QKI, SLC20A1, ST14, TPD52L1,
	TWIST1, VIM;
	**miR-203**: ARHGAP32, CDH1, ENSA, FAM84B,
	OVOL1, PARD6B, TC2N, TPD52L1, VIM;
	**miR-301a**: AP1S2, BICD2, ERMP1, ESRP2, IRF6,
	MAL2, MAP1B, MAP7, PRRG4, SLC20A1, TRAF4,
	TTL, TWIST1;
	**miR-301b**: AP1S2, BICD2, MAP1B, PRRG4, TRAF4,
	TTL;
	**miR-32**: BICD2, QKI, RAB8B, RBM47, RNF43,
	SACS, TWIST1, VIM;
	**miR-429**: GRHL1, QKI, TWIST1;
	**miR-590-3p**: CDS1, DSP, ELOVL5, MAP1B, MRPL49,
	PARD6B, RAB8B, RBM47, SACS, SLC20A1;
	**miR-7**: ARHGAP32, DSC2, DSP, EPN3, ESRP1,
	F11R, FAM83H, FAM84B, GRHL1, GSR, LPAR2,
	MAP1B, MYO5B, PARD6B, PLS1, QKI, RAB11FIP4,
	S100A14, SACS, SLC29A2, TRAF4;
C2N65	**miR-141**: TMC5;
	**miR-148b**: EFNA1, MUC13, TBC1D30, TSKU;
	**miR-200a**: PLEKHF2, TMC5, TSKU;
	**miR-200b**: EFNA1, TACSTD2;
	**miR-200c**: EFNA1; **miR-301a**: PLEKHF2, TSKU;
	**miR-301b**: PLEKHF2; **miR-32**: PALM2-AKAP2;
	**miR-429**: EFNA1; **miR-7**: ANGEL1, LPAR2, TSKU;
C3N65	**miR-101**: AP1S2, BICD2, CLDN4, DLG3, MSN,
	RAB8B, SACS, SLC29A2, TST;
	**miR-106b**: BICD2, BMP4, DSP, ESRP1, F11R,
	GIPC2, KIAA1522, MAP7, MCF2L, MPZL2, MYO5B,
	OSTM1, PARD6B, PLS1, RAB8B, RBM47, S100P,

### Functional enrichment analysis of the COREs

**Fig. 2:**
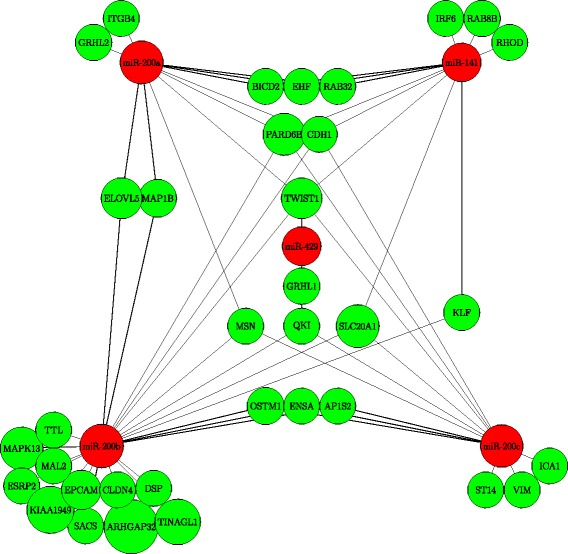
Confirmed interactions for miRNAs of the *miR-200* family included in the top CORE ‘C1N65’ obtained from the NCI60. Red nodes are miRNAs, and green nodes are experimentally confirmed target mRNAs

A CORE consists of a group of miRNAs and a group of mRNAs, in which the individual interactions between miRNAs and mRNAs play a vital role. To demonstrate the effectiveness of *DICORE*, we identified the interactions in the obtained COREs and compared them with the experimentally confirmed interactions found in the ‘ground-truth’ databases. The confirmed interactions of the top COREs identified from the NCI60 dataset for *η*=0.65 are summarized in Table 4. The confirmed interactions for the miRNAs in the *miR-200* family included in the top CORE ‘C1N65’ are illustrated in Fig. 2 using an example CORE, where red nodes are miRNAs and green nodes are experimentally confirmed target mRNAs. The higher number of confirmed interactions demonstrated the effectiveness of *DICORE*.

**Table 4:** Confirmed interactions in COREs from the NCI60 for *η*=0.65 *(Continued)*

	SACS, SLC29A2, TBC1D30
	**miR-18a**: BICD2, BMP4, ELOVL5, ESRP1, INADL,
	MAP1B, MARK2, PARD6B;
	**miR-18b**: BICD2, ESRP1, INADL, MAP1B, SCNN1A;
	**miR-30e**: ABHD11, ANPEP, DSP, ELOVL5, FAM84B,
	GCNT3, GRHL2, ITGB4, MANSC1, MCF2L, OVOL1,
	PARD6B, PLS1, PPL, QKI, RAB32, RAB8B,
	SACS, SLC20A1, TC2N, VIM;
	**miR-96**: CDH1, CEP170, DSP, MAL2, PARD6B,
	RHOD, SLC20A1, TUBA1A, VIM;
C4N65	**miR-200b**: VIPR1; **miR-7**: RABGAP1L
C5N65	**miR-106b**: TBC1D30; **miR-18a**: SLC12A2;
	**miR-30e**: MOSC1, SLC12A2, TACSTD2;
	**miR-96**: EFNA1, ERBB3, PRPS1, TSKU;

miRNAs are highlighted in bold-face texts

We got similar higher experimentally confirmed interactions for top COREs identified from BR and MCC datasets. The experimentally confirmed interactions for top COREs identified from the three datasets are listed in Additional file 3.

### Pathway analysis of the COREs

A *biological pathway* is a group of genes that participate in a particular biological process to perform certain functionality in a cell. To find the controlling factors of a disease, it is meaningful to study the genes by considering their pathway information.

We used the GeneCodis [39] online tool at [40] to conduct pathway enrichment analysis of the COREs with the focus on significant Kyoto Encyclopedia of Genes and Genomes (KEGG) [41] pathways (adjusted *p*-value <0.05). We selected the top COREs ‘C1N60’, ‘C1B60’, and ‘C1M45’ discovered from the NCI60, BR, and MCC datasets, respectively for the analysis, and the top 7 enrichment KEGG pathways annotated with the COREs are listed respectively in Tables 5, 6 and 7 with their *p*-values, where the *p*-values are adjusted by Benjamini-Hochberg (BH) method. As shown in the tables, all the COREs are significantly associated with the KEGG pathway: *Pathways in cancer*. Since the three datasets are all cancer datasets, the results demonstrate that the identified COREs are closely related to the biological conditions of their respective datasets.

**Table 5:** Top 7 enrichment KEGG pathways for CORE ‘C1N60’ from the NCI60 for *η*=0.60

No	KEGG Pathways	*p*-value
1	Tight junction	9.28E–08
2	Arrhythmogenic right ventricular	5.73E–04
	cardiomyopathy (ARVC)	
3	Glutathione metabolism	3.40E–03
4	Leukocyte transendothelial migration	4.84E–03
5	Axon guidance	8.35E–03
6	Pathways in cancer	1.01E–02
7	Endocytosis	1.44E–02

**Table 6:** Top 7 enrichment KEGG pathways for CORE ‘C1B60’ from the BR for *η*=0.60

No	KEGG Pathways	*p*-value
1	Inositol phosphate metabolism	2.44E–02
2	Complement and coagulation cascades	3.01E–02
3	Regulation of actin cytoskeleton	3.42E–02
4	Phosphatidylinositol signaling system	3.59E–02
5	Pathways in cancer	4.05E–02
6	ECM-receptor interaction	4.44E–02
7	Prostate cancer	4.59E–02

**Table 7:** Top 7 enrichment KEGG pathways for CORE ‘C1M45’ from the MCC for *η*=0.45

No	KEGG Pathways	*p*-value
1	Vascular smooth muscle contraction	3.85E–08
2	Oocyte meiosis	6.07E–04
3	Complement and coagulation cascades	2.37E–03
4	Adherens junction	2.41E–03
5	Long-term depression	2.52E–03
6	Pathways in cancer	3.05E–03
7	Tight junction	4.46E–03

Again, we used GeneGo Metacore [42] from GeneGo Inc. to identify the pathways previously discovered in the literature that involve the mRNAs in the identified top COREs. Table 8 shows the first 10 pathways as well as some other related pathways identified for another top CORE ‘C1N65’ from the NCI60 dataset. It confirms that ‘C1N65’ is highly relevant to the biological condition of the dataset. For instance, pathways number 1, 8, 11, 14 and 20 are direct pathways of the development of EMT, and others are important pathways involved in the process of EMT. Moreover, pathway number 1 includes total 12 members, of which 7 were identified in ‘C1N65’.

**Table 8:** GeneGo mapped pathways for CORE ‘C1N65’ from the NCI60 for *η*=0.65

No	Pathway maps	*p*-value
1	Development_miRNA-dependent inhibition of EMT	3.38E–12
2	Cytoskeleton remodelling_Keratin filaments	2.41E–11
3	Cell adhesion_Endothelial cell contacts by junctional mechanisms	1.03E–07
4	Cell adhesion_Tight junctions	8.05E–07
5	Cell adhesion_Gap junctions	1.54E–04
6	Development_Neural stem cell lineage commitment (schema)	3.92E–04
7	Cell cycle_Role of 14–3–3 proteins in cell cycle regulation	1.03E–03
8	Hypoxia–induced EMT in cancer and fibrosis	2.90E–03
9	LRRK2 in neurons in Parkinson’s disease	3.39E–03
10	G–protein signaling_RhoA regulation pathway	3.69E–03
11	Development_TGF– *β*–dependent induction of EMT via SMADs	4.01E–03
14	Development_TGF– *β*–dependent induction of EMT via MAPK	9.18E–03
20	Development_Regulation of EMT	2.11E–02

The pathway enrichment analysis has clearly justified the use of CCA in ranking the COREs, as the top ranked COREs show higher biological significance, and represent the given datasets. The detailed information of significant pathways identified from the three datasets is summarized in Additional file 4.

### Implication of the COREs in cancer

Since all of the input datasets included the expression profiles of miRNAs and genes associated with cancer samples, it is expected that the COREs identified from those datasets are to be related to cancer. To verify this, we used a cancer miRNA benchmark dataset of 147 miRNAs from a review article of [43]. Each of these miRNAs was reported in the literature to be dysregulated in one or more cancers.

The NCI60 dataset has 14 miRNAs from the benchmark, and except for *miR-205*, rest 13 are included in the top COREs. Both the top COREs ‘C1N60’ and ‘C1N65’ from the NCI60 dataset included 9 of the 14 miRNAs (namely *miR-141*, *miR-148b*, *miR-200a*, *miR-200b*, *miR-200c*, *miR-203*, *miR-301a*, *miR-32*, *miR-7*), which are associated with different cancers like Glioblastoma, Prostate, Lung, Bladder, Colon, Breast, Esophageal, Colorectal, Hepatocarcinoma, Ovarian, squamous cell carcinoma of tongue (SCCT), and Pancreatic.

Again, among these 147 miRNAs, 34 miRNAs are relevant to breast cancer. The BR dataset has 7 miRNAs out of these 34, of which 4 are identified in the top COREs.

On the other hand, the MCC dataset has 49 miRNAs out of the benchmark 147 miRNAs. The only CORE for *η*=0.60 included 9 miRNAs, of which 8 are from the benchmark. These miRNAs are involved in verified association with breast cancer (*let-7d*, *miR-98*, *miR-101*), ovarian cancer (*let-7c*, *let-7d*, *miR-100*, *miR-126*, *miR-99a*), prostate cancer (*let-7c*), Burkitt Lymphoma cancer (*let-7c*), pancreatic cancer (*let-7d*, *miR-100*), bladder cancer (*miR-100*, *miR-195*, *miR-99a*), SCCT cancer (*miR-100*, *miR-195*, *miR-99a*), lung cancer (*miR-101*, *miR-126*), cervical cancer (*miR-126*), colon cancer (*miR-126*), and hepatocarcinoma (*miR-126*) [43]. It is interesting to note that one of the important parts of the COREs identified from MCC, i.e. the *let-7* family has special characteristics and mechanisms of tumor suppressor activity [44, 45].

### Targets prediction for miRNAs of the COREs

**Fig. 3:**
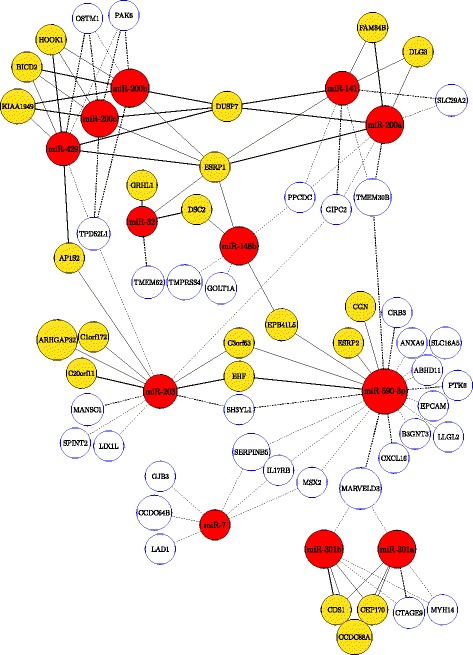
Predicted interactions for miRNAs included in the top CORE ‘C1N65’ obtained from the NCI60. Red nodes are miRNAs, yellow and white nodes are predicted (conserved) targets and poorly conserved targets of conserved miRNA families, respectively. Solid lines and dashed lines are used to represent links between miRNAs and their conserved targets and poorly conserved targets, respectively. The interactions are predicted by both *DICORE* and TargetScan

In this section, we report a set of novel miRNA-mRNA interactions for further experiments. These miRNA-mRNA interactions identified by *DICORE* are the predicted targets of conserved miRNA families in TargetScan v6.2 [4, 46]. Fig 3 visualizes the predicted interactions in a model interaction representation of the CORE ‘C1N65’, where red nodes are miRNAs, yellow nodes are conserved target mRNAs, and white nodes are poorly conserved target mRNAs. Predicted (conserved) interactions for top COREs from the three databases are given in Additional file 5.

### Comparison with other methods

We summarize here the comparison study of the result of *DICORE* with the results of a few recent methods *Mirsynergy* [27], *SNMNMF* [25], and *PIMiM* [47] reported in [27]. We obtained the same ovarian cancer (OVC) dataset processed in [25]. The original miRNA and gene expression profiles for 385 ovarian cancer samples were downloaded from [48]. The expression dataset contains measurements of 559 miRNAs and 12456 mRNAs.

In case of performing a comparison study, our initial intention was to compare the result of *DICORE* with the results of two other methods, *Mirsynergy* [27] and *SNMNMF* [25] by applying them to the three cancer datasets (NCI60, BR and MCC) used for validating *DICORE*. However, both *Mirsynergy* and *SNMNMF* require as their input the gene-gene interactions (GGIs) derived from protein-protein interactions and transcription factor binding sites, which, according to [25] and [27], are to be obtained from the two datasets, BioGrid [49] and TRANSFAC [50]. Unfortunately, we could only manage to get the GGIs associated with the three cancer datasets from BioGrid. As a consequence, the results we obtained from *Mirsynergy* using these three cancer datasets were not good. Therefore to make a fair comparison with the two methods, we apply our method to the dataset (the OVC dataset) on which *Mirsynergy* and *SNMNMF* have had their results reported in the literature.

Similar to the setting used in [27], we used the absolute values of only negative interaction weights of *W* and same pair of values for the density thresholds, and set 2 for the penalty value in calculating cohesiveness scores. In addition, we set *Θ*=(*θ*_*m*_,*θ*_*g*_)=2 for both groups of miRNAs and mRNAs due to the requirement for calculating CCA, as CCA can not be applied on groups having less than 2 components.

Table 9 shows a summary of the performance of the four methods. *DICORE* identified 56 modules with an average of 8.3 miRNAs and 43.83 mRNAs per module for *η*=0.35. The average strength of collective relationships is 0.61 in terms of canonical correlation among the groups. Furthermore, for *η*=0.30, *DICORE* got 102 modules with 11.23 miRNAs and 73.22 mRNAs per module, and having average strength of 0.60. The average number of mRNAs identified by *Mirsynergy* is too small compared to other methods. However, average number of mRNAs identified by *DICORE* is reasonable.

**Table 9:** Performance of *DICORE*, *Mirsynergy*, *SNMNMF*, and *PIMiM*

Method	*#* *C*	$\overline {miR}$	$\overline {mR}$
*DICORE*	56	8.30	43.83
*Mirsynergy*	84	4.76	7.57
*SNMNMF*	49	4.12	81.37
*PIMiM*	40	4.70	67.80

We report here two interesting modules. Firstly, the module or CORE ‘C9O35’ consists of 4 miRNAs, namely miR-29c*, *miR-29a*, *miR-29b*, and *miR-29c* from the same *miR-29* family. The human *miR-29* family of miRNAs is known to be associated with ovarian cancer [43, 51]. The pathway analysis of this module also shows association with cancer (see Table 10).

**Table 10:** Top enrichment KEGG pathways for ‘C9O35’ from the OVC for *η*=0.35

No	KEGG Pathways	*p*-value
1	Basal cell carcinoma	3.85E–08
2	Arginine and proline metabolism	0.0298836
3	Glutathione metabolism	0.0398112
4	Pathways in cancer	0.0426415
5	Cell cycle	0.0495754

The module or CORE ‘C17O35’ included 16 miRNAs and 74 mRNAs. The module has miRNAs, namely *miR-17*, miR-19b-1*, *miR-19b*, *miR-19a*, *miR-18b*, *miR-18a*, miR-20a*, *miR-20a*, *miR-20b* from the polycistronic miRNA cluster *miR-17-92*, located in chromosome 13. They are considered to act as a tumor suppressor for ovarian cancer in some circumstances [52]. Furthermore, the pathway analysis of this module also illustrates association with cancer (see Table 11).

**Table 11:** Top enrichment KEGG pathways for ‘C17O35’ from the OVC for *η*=0.35

No	KEGG Pathways	*p*-value
1	Pathways in cancer	0.00679353
2	MAPK signaling pathway	0.0136845

The final module structure of *Mirsynergy* is heavily depended on the initial clustering of miRNAs and the prior knowledge of gene-gene interactions. If *Mirsynergy* gets *c* clusters of miRNAs in the first stage, finally it will produce at most *c* miRNA regulatory modules. On contrary, *DICORE* separately performs clustering of miRNAs and mRNAs based on their functional interactions with mRNAs and miRNAs, respectively. This allows two distinct groups of mRNAs functioning differently to be part of different modules despite the fact that they are interacting with the same group of miRNAs. Furthermore, it also allows a group of miRNAs to interact with more than one group of miRNAs, which is common in biological sense.

## Related works

Several computational approaches had been proposed to discover MMRMs. The concept of MMRMs was introduced by [18] to denote groups of co-expressed miRNAs and their targets mRNAs. They drew a similarity between predicting MMRMs and mining frequent itemset by mapping the set of miRNAs and the set of target mRNAs to a frequent itemset and its cover, respectively. They proposed a prediction method adopting bipartite graphs to model binding structures of the miRNAs and mRNAs at the sequence level. However, prediction based on sequence may not be sufficient to correctly predict the complex interactions.

Improved versions of this method had been proposed which also take into account coherent expression patterns between miRNAs and mRNAs, or the (anti)-correlations measured between each pair of miRNAs and mRNAs [19, 21, 26]. Joung et al. [21] integrated expression profiles of miRNAs and mRNAs with sequence information by using a biclustering approach. The approach reduced false discovery rate significantly. A rule based method was utilized by Tran et al. [19] based on the assumption that miRNAs and mRNAs of a module have similar expression patterns. However, these existing methods for discovering MMRMs suffered from several limitations. For example, Peng et al. [26] proposed a sequential integrative method based on enumerating maximal bicliques in a combined miRNA-gene network. Their method was sensitive to noise in the data, and produced too many star structures (one miRNA, many genes) which were not usable to explore miRNA combinatorial regulation.

The *functional* MMRMs (FMMRMs) are associated with MMRMs with specific biological conditions. For FMMRMs discovery, [22] and [20] proposed different methods at around the same time. Joung and Fei [22] proposed an unsupervised method which applied the author-topic model [53, 54] in bioinformatics. The method used the expression profiles of miRNAs and the putative miRNA target information, without considering the expression profiles of miRNAs. As the miRNA target information is predicted at the sequence level, it encountered similar difficulty of [18] in explaining regulation pattern of miRNAs in their target genes in the identified modules. On the other hand, Liu et al. [20] proposed a supervised method which utilized association rule mining method by associating the reverse expression patterns of miRNAs and genes with biological conditions. However, they only considered down-regulation patterns.

In order to discover FMMRMs, Liu et al. [23] applied another probabilistic graphical model, correspondence Latent Dirichlet Allocation (Corr-LDA) [55], that had been applied to automatic image annotation with caption words. By associating topics to functional modules, images to miRNAs, and words to mRNAs, respectively, the method was applied to a mouse model dataset for human breast cancer research. The method simultaneously identified FMMRMs using the expression profiles of both miRNAs and genes, with or without using target relationships between miRNAs and mRNAs. The Corr-LDA was extended and applied to identify functional regulatory module, and each module corresponds to a particular biological function. In the model, each function was represented as a latent topic, and the numeric values of expression data were converted to the counts of expression events, similar to the counts of words in a documents. Another similar semi-supervised method based on a probabilistic model which is closely associated with the Latent Dirichlet Allocation [56] was proposed in [24]. The idea of extracting topics with caption words to FMMRMs discovery by mapping topics to functional modules, documents to samples, and words to mRNAs, respectively.

The main drawback of these methods is that they did not consider the collective relationships in identifying the modules, which result in regulatory modules that may not quite correct modeling of the real biological systems. Recently Karim et al. [28] came up with the idea of collective group relationships, and proposed a method to explore miRNA-mRNA regulatory relationships. They integrated two complementary approaches associated with relationships of complex systems, namely graph mining and CCA to discover collective relationships with both quantitative and qualitative information. However, the proposed method considered unweighted graph, which are prone to make computational inaccuracy due to the approximation of many interaction weights to either 1 (interaction) or 0 (no interaction). Recently Li et al. [27] proposed a clustering algorithm, *Mirsynergy* to detect synergistic miRNA regulatory modules. They used mRNA and miRNA expression profiles, target site information and gene-gene interactions for ovarian, breast, and thyroid cancers from TCGA [57] and obtained significantly higher enrichment than existing methods. However, it partially used collective relationships in stage 1, and but in stage 2 depended on the prior knowledge of confirmed gene-gene interactions.

This paper presents a novel method that discover MMRMs by considering the collective relationships as the driving force in identifying the miRNA-mRNA regulatory relationships. Furthermore, it uses the idea of ranking the identified modules by the quantitative measure of the strength of the collective relationships between the groups in group pairs.

## Conclusion

In this paper, we have used the notation of CORE, and proposed a computational framework *DICORE* to discover MMRMs. The central idea of *DICORE* is to consider the collective group relationship, and discover both the groups and collective relationships simultaneously. We have applied a greedy-based overlapping clustering approach adapted from *ClusterONE* [29] to group miRNAs and mRNAs separately based on their collective interactions with mRNAs and miRNAs respectively, and integrate CCA in order to enrich the identification of groups with both structural link information and strength of collective relationships. We have experimented on three real-world biological datasets. The experimental results have demonstrated that the proposed method *DICORE* is able to reveal correct group information with structural link information and the strength of collective relationships, and provide useful insights into the structure and functionality of the miRNA-mRNA regulatory relationships in MMRMs.

The proposed framework has also opened a few interesting research windows for further investigation. Instead of using Pearson correlation coefficient to calculate the interaction weights matrix, other approaches including statistical methods like maximal information coefficient [58], regression techniques like Lasso [59], causal inference method like IDA [31] can be applied. Considering the context of the datasets, any of the individual methods or an ensemble method [31] can be tested and reported. Furthermore, the strength of the collective interactions can be determined by applying other similar mathematical models to capture all possible association between two sets of variables. Another interesting future work will be to apply the framework to discover MMRMs from datasets obtained under different biological conditions.

## Additional files

Additional file 1
**Differential expression profiles of miRNAs and mRNAs for the NCI60, BR and MCC datasets.** (XLSX 2785 kb)

Additional file 2
**Three separate ‘ground-truth’ databases for the NCI60, BR and MCC datasets, respectively.** (XLSX 227 kb)

Additional file 3
**Experimentally confirmed interactions of the top COREs identified in the NCI60, BR and MCC datasets.** (XLSX 33.5 kb)

Additional file 4
**The significant pathways of top group pairs identified in the NCI60, BR and MCC datasets.** (XLSX 21.9 kb)

Additional file 5
**Predicted miRNA-mRNA interactions identified by** ***DICORE*** **in the NCI60, BR and MCC datasets.** (XLSX 14.0 kb)
